# 

**Published:** 1916-12

**Authors:** 


					﻿TEXAS
Medical Journal
				

